# Infrequent Transmission of Monovalent Human Rotavirus Vaccine Virus to Household Contacts of Vaccinated Infants in Malawi

**DOI:** 10.1093/infdis/jiz002

**Published:** 2019-01-24

**Authors:** Aisleen Bennett, Louisa Pollock, Khuzwayo C Jere, Virginia E Pitzer, Benjamin Lopman, Umesh Parashar, Dean Everett, Robert S Heyderman, Naor Bar-Zeev, Nigel A Cunliffe, Miren Iturriza-Gomara

**Affiliations:** 1 Malawi-Liverpool-Wellcome Trust Clinical Research Programme, Blantyre; 2 Centre for Global Vaccine Research, Institute of Infection & Global Health, Liverpool; 3 Medical Laboratory Department, College of Medicine, University of Malawi, Blantyre; 4 Department of Epidemiology of Microbial Diseases, Yale School of Public Health, Yale University, New Haven, Connecticut; 5 Department of Epidemiology, Rollins School of Public Health, Emory University, Atlanta, Georgia; 6 Centers for Disease Control and Prevention, Atlanta, Georgia; 7 MRC Centre for Inflammation Research, University of Edinburgh, United Kingdom; 8 Division of Infection and Immunity, University College London, United Kingdom; 9 Department of Global Disease Epidemiology and Control, Johns Hopkins Bloomberg School of Public Health, Baltimore, Maryland; 10 National Institute for Health Research Health Protection Research Unit in Gastrointestinal Infections, University of Liverpool, United Kingdom

**Keywords:** rotavirus, vaccine, indirect effects, transmission, Malawi

## Abstract

Horizontal transmission of rotavirus vaccine virus may contribute to indirect effects of rotavirus vaccine, but data are lacking from low-income countries. Serial stool samples were obtained from Malawian infants who received 2 doses of monovalent human rotavirus vaccine (RV1) (days 4, 6, 8, and 10 after vaccination) and from their household contacts (8–10 days after vaccine). RV1 vaccine virus in stool was detected using semiquantitative real-time reverse-transcription polymerase chain reaction. RV1 fecal shedding was detected in 41 of 60 vaccinated infants (68%) and in 2 of 147 household contacts (1.4%). Horizontal transmission of vaccine virus within households is unlikely to make a major contribution to RV1 indirect effects in Malawi.

Programmatic introduction of rotavirus vaccines has led to substantial reductions in the global burden of severe rotavirus gastroenteritis; however, rotavirus vaccine effectiveness is significantly lower in low-income than in higher-income settings [1]. Rotavirus remains a major cause of severe gastroenteritis in African children, with an estimated 121 009 deaths from rotavirus gastroenteritis in sub-Saharan Africa in 2013 [2]. In this context, indirect effects of rotavirus vaccine, which have been well documented in high-income settings [3], could make an important contribution to overall vaccine impact. Mechanisms of indirect effects of rotavirus vaccine may include reduction in transmission of wild-type rotavirus and/or horizontal transmission of vaccine virus to contacts after excretion in stool of vaccinated infants.

Live oral rotavirus vaccines mimic natural infection, replicating in the gastrointestinal tract before being shed in stool. Several studies have demonstrated horizontal transmission of rotavirus vaccine virus from vaccinated infants to close contacts; reported transmission rates range from 0% to 18.8% [4–6]. The protective effects of horizontal transmission of vaccine virus to unvaccinated individuals has been well described for oral polio vaccine [7], but the role of such transmission in generating vaccine indirect effects for rotavirus is not established.

To our knowledge transmission of rotavirus vaccine virus has not previously been investigated in Africa, where—compared with higher-income countries—differences in host vaccine response, population structure, contact patterns, sanitation, and underlying comorbid conditions [1] could affect both vaccine virus shedding in vaccine recipients and susceptibility of close contacts to infection. We undertook a prospective cohort study in Malawi, a low-income African country with a high burden of rotavirus disease. The monovalent human rotavirus vaccine (RV1; Rotarix) was introduced in Malawi in 2012, with doses delivered at 6 and 10 weeks of age. Postimplementation effectiveness has been reported at approximately 60% [8]. We aimed to estimate the proportion of household contacts of RV1-vaccinated infants who subsequently shed vaccine-type virus.

## METHODS

### Study Design and Procedures

We conducted a prospective cohort study between April and August 2016. After informed consent, consecutive infants attending a government health center for routine vaccinations were recruited (index infants) together with their household contacts (children and adults).

Index infants had a prevaccine baseline stool sample collected at recruitment and 4, 6, 8, and 10 days after vaccination. Stool samples were collected from household contacts before administration of the first vaccine dose to the index infant and 8–10 days after administration of each vaccine dose. Sampling times were based on local pilot data and prior studies of infant vaccine virus shedding and assumed peak shedding at day 6, with 2–3 days allowed for transmission to and subsequent shedding in contacts [4, 5]. Maternal human immunodeficiency virus (HIV) status was extracted from hand-held government health records, or by maternal report when records were unavailable. HIV exposure was defined as an infant born to an HIV-infected mother.

Stool specimens were tested for rotavirus using VP6 semi-quantitative real-time reverse transcription polymerase chain reaction (qRT-PCR) [9] and vaccine virus–specific NSP2 qRT-PCR [10]. Assays were considered positive if the cycle threshold (Ct) value was <40. Samples positive by both NSP2 qRT-PCR and VP6 qRT-PCR were classified as positive for vaccine virus. Samples positive by VP6 qRT-PCR but negative by NSP2 qRT-PCR were assumed to represent wild-type rotavirus. In index infants, postimmunization vaccine virus shedding was defined as ≥1 confirmed postvaccination virus-positive sample.

We used χ^2^ tests to compare categorical variables and McNemar tests to compare paired proportions before and after exposure. The study was powered to detect a difference in the paired proportion of individuals shedding vaccine virus before and after exposure to a vaccinated infant, with 80% power and an α value of .05 determined with the McNemar test [11] and assuming a shedding frequency of 50% in recently vaccinated infants (based on pilot data in the same population). A sample of 60 infants (and their household contacts) was required in order to detect a postexposure increase in shedding of ≥30%; we planned to recruit 75 infants to allow for loss to follow-up. This study was approved by the University of Liverpool Research Ethics committee (Application numbers 000757 and 000758) and the Malawi College of Medicine Research Ethics Committee (Application numbers P.09/14/1623 and 1624).

## RESULTS

A total of 72 households were recruited. Of these, 69 households (69 index infants, 147 household contacts) completed follow-up. The median age of infants at recruitment was 6.1 weeks (range, 5.1 to 8.7 weeks). Of the 69 infants, all were exclusively breastfed, 29 (42%) were male, and 12 (17%) were HIV exposed. Of the HIV-exposed infants, 5 were HIV uninfected (PCR negative for HIV DNA) at 6 weeks of age; the HIV status of the other 7 exposed infants was unknown at the end of follow-up.

Owing to infrequent passing of stool in breastfed infants and logistic constraints, stool samples were not available for every vaccine-recipient infant at every collection time point. Among infants with a baseline stool sample, 1 of 33 samples (3%; 95% confidence interval [CI], 0.4%–20%) was weakly positive for vaccine virus (Ct value 38.6). Wild-type rotavirus was detectable at baseline in 5 of 33 samples (15%; 95% CI, 6%–33%), with a median Ct value of 38.5 (interquartile range [IQR], 37.9–38.6). Of 60 index infants who provided sufficient stool for analysis of both dose periods, 41 (68%; 95% CI, 55%–79%) had detectable vaccine virus shedding after either dose 1 or dose 2. Shedding was detected early, with first detection by day 6 in 25 of 29 infants (86%) who shed in the first dose period and 26 of 34 (76%) who shed in the second dose period (Table 1). Shedding density was low, with a peak shedding median Ct value of 32.2 (IQR, 29.9–35.8) after the first vaccine dose and 32.8 (30.5–35.5) after dose 2. The RV1 shedding patterns by dose period are provided in Supplementary Table 1.

**Table 1.** Proportion of Infants With RV1 Vaccine Virus Shedding After RV1 Immunization

Dose Period	Infants With RV1 Vaccine Virus Shedding, No./Total (%; 95% CI)				
	Day 4	Day 6	Day 8	Day 10	Overall
First	15/58 (26; 16–39)	18/61 (30; 19–42)	15/56 (27; 17–40)	12/65 (18; 20–30)	29/68 (43; 31–55)
Second	20/57 (35; 24–48)	21/60 (35; 24–48)	20/57 (35; 24–49)	17/55 (31; 20–45)	34/64 (53; 41–65)

Abbreviations: CI, confidence interval; RV1, monovalent human rotavirus vaccine.

Of 147 household contacts recruited (46 of 147 [31%] male; median age, 21 years; range, 0–41 years), 119 provided prevaccine stool samples, 138 provided samples after the first dose, 122 after dose 2, and 113 after both doses. Postvaccination samples were collected a median of 8 days (IQR, 8-8 days) after dose 1 and 8 days (8–11 days) after dose 2. Characteristics of household contacts are shown in Table 2. There were no reported episodes of gastroenteritis in household contacts during the follow-up period. No contacts shed vaccine virus before exposure to a vaccinated infant. A total of 2 of 113 (1.8%; 95% CI, 0.4%–6.9%) household contacts, from different households, shed vaccine virus after exposure to a vaccinated infant; these contacts included 1 sibling (aged 3 years, HIV status unknown) and 1 mother (aged 34 years, HIV negative).

**Table 2.** Characteristics of Households and Household Contacts

Characteristic	Households or Contacts, No. (%; 95% CI %)
Households (n = 69)	
Other children aged <5 y in household	23 (33; 23–46)
Toilet shared with other households	54 (78; 67–87)
Typical time required to access water, mins	
<5	30 (44; 32–56)
5**–**30	36 (52; 40–64)
>30	3 (4; 1–13)
Typical source of domestic water	
Well	1 (1.5; 0.2–10)
Borehole	4 (6; 2–15)
Tap shared with other households	50 (72; 60–82)
Private tap to house	14 (20; 12–32)
Household contacts (n=147)	
Age, y	
<5	20 (14; 9–20)
5**–**15	30 (20; 15–28)
15**–**45	97 (66; 58–73)
Male sex	46 (31; 24–39)
HIV infected	13/77 (17; 10–27)
Relationship to infant	
Mother	69 (47; 39–55)
Father	20 (14; 9–20)
Other adult relative	4 (3; 1–7)
Child relative	54 (37; 29–45)
Prior rotavirus vaccine in contacts aged <5 y	8/17 (44; 24–72)
Sleeps in same room as vaccinated infant	108 (73; 66–80)
Sleeps in same bed as vaccinated infant	91 (62; 54–69)
Time spent with child	
All day	83 (56; 48–64)
Half day	42 (29; 22–36)
Evening only	22 (15; 10–22)
Primary caregiver for vaccinated infant	71 (48; 40–56)
Responsible for changing diaper of vaccinated infant	
Never	75 (51; 43–59)
Sometimes	3 (2; 1–6)
Always	69 (47; 39–55)

Abbreviations: CI, confidence interval; HIV, human immunodeficiency virus.

Wild-type rotavirus was detectable in at least 1 stool sample in 35 of 147 household members (24%; 95% CI, 18%–31%). Shedding was low level (median Ct value, 39; IQR, 36–39). There was no difference in the frequency of wild-type shedding with age: 6 of 20 children <5 years old (30%) shed wild-type virus, compared with 29 of 127 (23%) >5 years old (*P* = .484). Viral loads in wild-type infections were too low to allow genotyping.

## DISCUSSION

To our knowledge this is the first study to investigate rotavirus vaccine virus transmission in households in a low-income setting. We found a very low rate of horizontal transmission of rotavirus vaccine virus to household contacts, despite a high frequency of vaccine virus shedding among vaccinated infants. The low transmission rates of vaccine virus observed in our study are consistent with a study in a foster home in Japan, which reported no horizontal transmission (determined by means of RT-PCR in serial samples) among 22 unvaccinated infant contacts of 4 vaccinated infants [5]. Horizontal transmission rates to placebo recipients were also low (0%–3%) in several prelicensure vaccine trials from high- and middle-income countries [6], although transmission to community contacts might be expected to be lower than transmission within households.

Reported rates of horizontal transmission were much higher in a clinical trial in the Dominican Republic, in which twins were randomized to receive either RV1 or placebo [4]. Vaccine virus fecal shedding was detected in 15 of 80 placebo recipients (18.8%), although 5 of 15 transmission events (33%) preceded or coincided with detectable shedding in the vaccine recipient and may have been false-positives. A higher transmission rate between twins might also be expected because of lower preexisting immunity to rotavirus (owing to very young age) and very close contact with the vaccinated infant. In a mother-infant study from South Africa, mothers of vaccinated infants were noted to have higher antirotavirus immunoglobulin A titers after vaccination of their infants [12]. The authors suggested that this could imply horizontal transmission, but infant fecal shedding was not measured and a rise secondary to maternal wild-type infection could not be excluded. However, it is possible that in countries with higher vaccine efficacy than Malawi, such as South Africa, vaccine virus shedding in infants may be higher and horizontal transmission effects could occur.

Our study has several strengths. First, prior studies have reported vaccine virus transmission only to other infants, which in the context of universal rotavirus immunization is perhaps less relevant to indirect vaccine effects than transmission to older children and adults. Second, our study used qRT-PCR to detect vaccine virus shedding. The majority of previous studies have used enzyme immunosassay or cell culture, both of which are considerably less sensitive than qRT-PCR. Finally, to our knowledge, this is the only study of rotavirus vaccine virus transmission conducted in a setting with high background HIV prevalence. Malawi has an HIV prevalence of 13% in women of childbearing age; HIV has been associated with prolonged rotavirus shedding [13], and HIV-infected household contacts could be more susceptible to vaccine virus infection. The very low frequency of vaccine virus transmission observed in the current study argues against any increased risk of transmission associated with household HIV exposure; however, data from a larger cohort are required.

A limitation of our study is the relatively sparse sampling of household contacts owing to logistical constraints, making it possible that transient very early or late shedding episodes were not detected. However, shedding of vaccine virus occurred early in vaccinated infants; vaccine virus was detected by day 6 after vaccination in about 80% of infants with fecal shedding. Furthermore, the molecular detection methods used are highly sensitive even for very low-level viral shedding. In the Dominican study, most transmission events occurred within the first 10 days after immunization [4], and our study findings are also corroborated by those from the study in Japan, which sampled at multiple time points [5]. An additional limitation of our study population is that there were relatively few young children or infants among our household contacts. Older children and adults are more likely to be partially immune to rotavirus, which may contribute to the observed low frequency of horizontal transmission. Our findings may represent a minimum estimate of horizontal transmission, but given the very small number of transmission episodes identified, it seems unlikely that the true frequency should be substantially higher.

In contrast to the low frequency of vaccine virus shedding among household members, wild-type rotavirus shedding in household members was common (24%), consistent with previous local data [14]. Wild-type rotavirus shedding in household members is most likely a result of transmission from a symptomatic child. Although no symptomatic cases were recorded at the time of sampling, shedding in case patients and their contacts can be prolonged after an episode of rotavirus diarrhea. Possible explanations for the difference in transmission rates of wild-type versus vaccine virus could include differences in viral load and symptoms in symptomatic children compared with vaccine recipients. Viral load is much higher in symptomatic children: in a previous Malawian study [14], children with rotavirus gastroenteritis had a median Ct value of 19 compared with a median of 32 for vaccine virus shedding in this study. The importance of symptoms in transmission is demonstrated by a study from Ecuador, which identified rotavirus infection in 55% of household contacts of symptomatic index case patients, compared with 2% of household contacts of healthy control children, and found an association between symptom severity and risk of transmission [15].

In conclusion, this study identified very little horizontal transmission of vaccine virus to household contacts in urban Malawi, despite high background HIV prevalence, crowded living conditions, and poor sanitation. Horizontal transmission of vaccine virus seems unlikely to be a major contributing factor to indirect vaccine effects in this setting.

## Supplementary Data

Supplementary materials are available at *The Journal of Infectious Diseases* online. Consisting of data provided by the authors to benefit the reader, the posted materials are not copyedited and are the sole responsibility of the authors, so questions or comments should be addressed to the corresponding author.

Supplementary Data

## Notes

***Acknowledgments.*** We thank all infants and their families who participated and all members of the RotaRITE study team. We are grateful for the support of the Malawi Ministry of Health and clinical staff at the recruitment sites.

***Disclaimer.***The findings and conclusions in this report are those of the authors and do not necessarily represent the official position of the US Centers for Disease Control and Prevention. M. I. G. is affiliated with the National Institute for Health Research Health Protection Research Unit in Gastrointestinal Infections at University of Liverpool in partnership with Public Health England, in collaboration with University of East Anglia, University of Oxford, and the Quadram Institute. M. I. G. is based at the University of Liverpool. The views expressed are those of the authors and not necessarily those of the National Health Service, the National Institute for Health Research, the Department of Health, or Public Health England.

***Financial support.*** This study was supported by Wellcome Trust (clinical PhD fellowship grants 102466/Z/13/A to A. B. and 102464/Z/13/A to L. P., program grant 091909/Z/10/Z, and training fellowship grant 201945/Z/16/Z to K. C. J.), the Malawi-Liverpool-Wellcome Trust Clinical Research Programme (Wellcome Trust core grant strategic award 101113/Z/13/Z), and the US National Institutes of Health (grant R01-AI112970 to V. E. P.). The authors received no financial support or other form of compensation related to the development of the manuscript.

***Potential conflicts of interest.*** K. C. J. and N. B. Z. have received research grant support from GlaxoSmithKline Biologicals for work on rotavirus vaccines. N. A. C. has received research grant support and honoraria for participation in rotavirus vaccine advisory board meetings from GlaxoSmithKline Biologicals. M. I. G. has received research grant support from GlaxoSmithKline Biologicals and Sanofi Pasteur MSD for work on rotavirus. All other authors report no potential conflicts. All authors have submitted the ICMJE Form for Disclosure of Potential Conflicts of Interest. Conflicts that the editors consider relevant to the content of the manuscript have been disclosed.

Presented in part: African Rotavirus Symposium, Lilongwe, Malawi, 28th–30th May 2017; European Rotavirus Biology Meeting, Cork, Ireland, 18th–21st June 2017.
