# Supplementary material for: Infrequent Transmission of Monovalent Human Rotavirus Vaccine Virus to Household Contacts of Vaccinated Infants in Malawi
Source: J Infect Dis. 2019 Jan 24;219(11):1730–4. doi: 10.1093/infdis/jiz002 (PMC6500552; doi:10.1093/infdis/jiz002)
Supplement: Supplementary Data [file jiz002_suppl_supplementary_data.docx]

**Supplementary data**

Supplementary Table 1: RV1 shedding patterns by dose period

|  | RV1 shedding  2^nd^ dose period | No shedding  2^nd^ dose period | Total |
| --- | --- | --- | --- |
| RV1 shedding  1^st^ dose period | 14 | 11 | 25 (39%) |
| No shedding  1^st^ dose period | 20 | 19 | 39 (61%) |
| Total | 34 (53%) | 30 (47%) | 64 |

11/64 (17%) of infants shed RV1 following the 1^st^ dose of RV1 only

20/64 (31%) of infants shed RV1 following the 2^nd^ dose of RV1 only

14/64 (22%) of infants shed RV1 following both doses of RV1

19/64 (30%) of infants did not shed RV1 after either dose.

Shedding of RV1 in the first dose period did not predict shedding in the second dose period:

RR 1.1, 95% CI 0.67-1.7, p=0.71
